# Late Local Recurrence and Metastasis in Soft Tissue Sarcoma of the Extremities and Trunk Wall: Better Outcome After Treatment of Late Events Compared with Early

**DOI:** 10.1245/s10434-021-09942-8

**Published:** 2021-04-16

**Authors:** Arvid von Konow, Iman Ghanei, Emelie Styring, Fredrik Vult von Steyern

**Affiliations:** Department of Orthopedics, Department of Clinical Sciences Lund, Lund University, Skåne University Hospital, Lund, Sweden

## Abstract

**Background:**

Approximately 80% of soft tissue sarcoma (STS) recurrences, local and metastatic disease, are diagnosed within the first 3 years after primary diagnosis and treatment. Recurrences, however, can present after a longer period of remission. Our goal was to identify factors that may predict the risk of late recurrence.

**Methods:**

We identified 677 patients with STS of the extremities and trunk wall from a population-based sarcoma register. Of these, 377 patients were alive and event-free at 3 years and were included for analysis of possible risk factors for late recurrence.

**Results:**

Fifty-five of 377 (15%) patients developed late recurrence: 23 local recurrence, 21 metastasis, and 11 both manifestations. With R0 wide surgical margin as reference, R0 marginal (hazard ratio [HR] 2.6; *p* = 0.02) and R1 (HR 5.0; *p* = 0.005) margins were risk factors for late local recurrence. Malignancy grade (HR 8.3; *p* = 0.04) and R0 marginal surgical margin (HR 2.3; *p* = 0.04) were risk factors for late metastasis. We could not find a statistically significant correlation of late recurrence with many of the generally known risk factors for local recurrence and metastasis in STS. Outcome after treatment of late recurrences was better compared with outcome after treatment of early events.

**Conclusions:**

Late recurrences, albeit relatively rare, do occur. Outcome after treatment was good compared with outcome after early events. Long surveillance of all patients with high-grade STS, especially if R0 wide surgical margin is not achieved in the primary treatment, appear to be well justified.

**Supplementary Information:**

The online version contains supplementary material available at 10.1245/s10434-021-09942-8.

Soft tissue sarcomas (STS) of the extremities and trunk wall constitute a heterogenous group of malignant tumors comprising more than 50 histological subtypes.^1^ In mixed series of STS the 5-year disease-free survival is approximately two-thirds, with specific metastasis-free and local recurrence-free survival rates of approximately 70% and 80%, respectively.^2^,^3^ Metastatic disease, most common to the lungs, is generally associated with a poor prognosis.^4^

Several prognostic factors that correlate with risk for local recurrence (LR) and distant metastasis (DM) have been identified, e.g., malignancy grade, size, tumor depth, surgical margins, certain histological subtypes, and tumor characteristics.^5^–^7^ Staging systems from *AJCC* and *UICC* include malignancy grade, tumor size, metastatic disease, or lymph node engagement but have limited value for treatment decisions in clinical practice.^5^,^6^,^8^–^10^ In Sweden, the SING system is used to identify high-risk tumors. The SING system includes assessment of tumor size, vascular invasion, tumor necrosis, and growth pattern in high-grade tumors.^11^–^13^

After primary treatment, follow-up surveillance includes physical examination and imaging of the chest. MRI of the primary tumor site is performed when indicated. The objective of standardized follow-up routines is to identify recurrences early in order to improve the chances of remission and long-term survival.^5^ ESMO and Swedish guidelines recommend follow-up for 10 years with intervals depending on malignancy grade.^5^,^14^ Approximately 80% of relapses after primary treatment occur within the first 3 years.^3^,^5^,^15^–^17^ Risk factors for late recurrence are not well studied, and it has been suggested that the prognostic value of known risk factors diminish over time.^18^–^21^

The purpose of this study was to investigate the STS recurrence pattern, LR and/or DM, over time and identify possible risk factors and predictors for late tumor recurrence. We have evaluated patients who were event-free and alive 3 years after primary diagnosis. Late recurrence often is defined as a recurrence diagnosed later than 5 years after the primary diagnosis and treatment.^19^,^20^,^22^ We have chosen 3 years as a cutoff, because we believe that it better reflects the recurrence pattern of STS (see Discussion). Prognostic factors for late LR and late DM, respectively, were evaluated with the goal to investigate whether it is possible to identify which patients would or would not benefit from a long surveillance.

## Methods

We identified adult patients (>18 years) with primary STS of the extremities and trunk wall, diagnosed between 1986 and 2014 in the Southern Sweden healthcare region (1.8 million inhabitants), using the national sarcoma quality register.^2^,^23^–^25^ Patients have prospectively been enrolled in the register, which has complete coverage compared with the national cancer register. Patients who were not surgically treated, patients with DM at diagnosis and certain histological subtypes (e.g., rhabdomyosarcoma, Ewing’s sarcoma of soft tissue, dermatofibrosarcoma protuberans, and atypical lipomatous tumor) were excluded. The register includes data on diagnosis, treatment, and outcome. In addition to register data, clinical charts were reviewed.

Patients were followed according to the Swedish and ESMO guidelines with physical examination and chest imaging. High-grade tumors were followed every third month for the first 3 years, twice per year up to 5 years, and once per year thereafter. Low-grade tumors were followed twice per year the first 5 years, and thereafter annually. Patients with first recurrence within 3 years were considered to have an early recurrence and patients with first recurrence at 3 years or later were considered to have a late recurrence. Patients with early recurrence were analyzed for reference purpose (supplementary tables).

Tumor size was determined as the largest diameter on histopathologic examination and was dichotomized at both 5 and 8 cm as these cutoffs are commonly used in other studies; 8 cm is used in the Swedish guidelines to identify high-risk tumors. Vascular invasion was defined as presence of tumor cells within any space with endothelial lining. Both vascular invasion and microscopic necrosis were classified as present or not and peripheral growth pattern as pushing or infiltrative.^11^,^12^ Tumors were classified as either superficial, if strictly located above an unengaged fascia, or deep-seated if the fascia was invaded or the tumor was located deep to the fascia. The FNCLCC histologic grading system is currently used in the clinical setting at our center. However, a large part of our series was graded using the Broders’ four-tiered system that was used before the French grading system was introduced.^26^ Low grade (grade 1 and 2) translates approximately to FNCLCC grade 1 and high grade (grades 3 and 4) to FNCLCC grade 2 and 3. Surgical margin was, in accordance with Swedish guidelines, classified as R0 wide if the tumor was surrounded by a 10-mm cuff of healthy fatty, muscular, or loose areolar tissue or an unengaged fascia.^27^,^28^ The margin was classified as R0 marginal if there was less than 10-mm margin or engagement of the fascia, as R1 (intralesional) if there was microscopic growth at the resection margin, or as R2 if there was macroscopic residual tumor.^29^,^30^ In cases with more than one surgical procedure performed for the primary tumor, the margin obtained at the last surgery was recorded as final margin.

The study was approved by the Lund University Ethics Committee and meet the guidelines of their responsible governmental agency.

### Statistical Analysis

Statistical analysis was performed using log-rank test or Cox proportional hazard model and survival data visualized using Kaplan–Meier survival analysis. Only variables that were statistically significant in univariable analysis were included in the multivariable analysis, because the number of events would not allow all variables to be included. A global significance test was calculated using likelihood ratio test. Time to event was calculated from the date of initial diagnosis to the recurrence date. Patients were censored at the last follow-up with no evidence of disease or at death without tumor. Survival was analyzed from diagnosis date or recurrence date to death or until censored. A *p* value < 0.05 was considered statistically significant. Statistical analysis was performed using Stata/IC 15.1 for Mac (StataCorp LLC, TX).

## Results

A total of 877 STS patients were identified of which 43 patients were excluded for not being surgically treated and 81 for having DM at the time of diagnosis. Another 61 cases were excluded due to certain histological subtypes, and 13 cases were lost to follow-up within 3 years. Two patients had insufficient data and were excluded. The remaining 677 patients were included in the study.

**Fig. 1:**
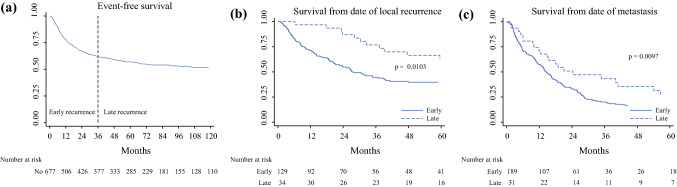
**a** Event-free survival from date of diagnosis of soft tissue sarcoma. **b** Survival in months from date of local recurrence for early and late recurrence (log-rank test). **c** Survival in months from date of metastatic disease for early and late recurrence (log-rank test)

Of these 677 patients, 250 (37%) had recurrence within the first 3 years from initial diagnosis: 60 patients had LR, 121 had DM, and 69 had both LR and DM. Another 50 patients without recurrence died within 3 years (mean age 81 years). The event-free survival for each year is presented in Table 1 and Fig. 1a. The 5-year overall survival (OS) was 63% (95% confidence interval [CI] 59–66), and the 10-year OS was 46% (95% CI 42–50) for these 677 patients.

**Table 1:** Event-free survival and cumulative number of events at the end of each interval from diagnosis of soft tissue sarcoma

Interval (years)	Event-free survival (95% CI)	No. at risk at beginning of interval	Cumulative number of events	Cumulative percentage of events	Recurrence hazard rate (SE)
0 to 1	78 (75–81)	677	147	48%	0.021 (0.0017)
1 to 2	67 (63–71)	506	217	71%	0.013 (0.0015)
2 to 3	62 (58–65)	426	250	82%	0.007 (0.0012)
3 to 4	59 (55–63)	377	265	87%	0.004 (0.0009)
4 to 5	57 (53–61)	333	277	91%	0.003 (0.0009)
5 to 6	55 (51–59)	285	287	94%	0.003 (0.001)
6 to 7	54 (50–58)	229	290	95%	0.001 (0.0007)
7 to 8	53 (49–57)	181	293	96%	0.002 (0.0009)
8 to 9	52 (47–56)	155	297	97%	0.002 (0.0012)
9 to 10	51 (47–55)	128	298	98%	0.001 (0.0007)
10 to 14	46 (41–51)	110	305	100%

### Event-Free at 3 Years

Patient and tumor characteristics for the 377 patients that were event-free and alive at 3 years from diagnosis are further described in Table 2. Seventy-seven percent of these tumors were high-grade, and the most common histological subtypes were undifferentiated pleomorphic sarcoma (21%), leiomyosarcoma (21%), and myxofibrosarcoma (18%). Less frequent subtypes (*n* < 10) were grouped for analysis. Of the STS located in the extremities, 96% underwent limb-sparing surgery for the primary tumor. The median age at diagnosis was 64 years (mean 60; range 18–96) with slight male predominance (57%). Median tumor size was 6 cm (mean 7; range 1–28). Ninety-two percent (95% CI 88.9–94.6) and 83% (95% CI 77.6–87.1) of these patients were still event-free at 5 and 10 years from diagnosis, respectively. The median follow-up time was 7.7 years (mean 9.1; range 3–26).

**Table 2:** Overview of patients and tumor characteristics

Characteristic	*n* = 377	%
Median age at diagnosis in years (mean; range)	64 (60; 18–96)	
Male	214	57
Female	163	43
Tumor depth (*n*, %)		
Superficial	175	47
Deep	196	53
Not available	6	
Tumor site (*n*, %)		
Upper trunk	29	8
Lower trunk	15	4
Shoulder	18	5
Upper arm	36	9
Elbow	11	3
Lower arm	30	8
Hand	5	1
Gluteal	23	6
Groin	11	3
Thigh	136	36
Knee	11	3
Lower leg	38	10
Foot	14	4
Median tumor diameter in cm (mean; range)	6 (7; 1–28)	
Tumor grade (*n*, %)		
1	24	7
2	55	16
3	71	20
4	201	57
Not available	26	
Vascular invasion		
Yes	38	12
No	273	88
Not available	66	
Necrosis		
Yes	167	52
No	157	48
Not available	53	
Growth pattern		
Pushing	20	19
Infiltrative	88	81
Not available	269	
Histologic subtype (*n*, %)		
Undifferentiated pleomorphic sarcoma	78	21
Myxofibrosarcoma	68	18
Liposarcoma	47	12
Leiomyosarcoma	81	21
Synovial sarcoma	19	5
Malignant peripheral nerve sheath tumor	18	5
Other type^a^	66	18
Surgical procedure (*n*, %)^b^		
Limb salvage	321	96
Amputation	12	4
Surgical margin (*n*, %)		
R0 wide	252	67
R0 marginal	109	29
R1	15	4
R2	0	0
Not available	1	
Radiotherapy		
Yes	129	34
No	248	66
Chemotherapy		
Yes	45	12
No	332	88

^a^Alveolar soft part sarcoma, clear cell sarcoma, epithelioid sarcoma, low-grade fibromyxoid sarcoma, solitary fibrous tumor, fibrosarcoma, unclassified sarcoma and angiosarcoma

^b^Not applicable to the 44 patients with tumors located to the trunk wall

Late recurrence was diagnosed in 55 (15%) of the 377 patients who were free from disease and alive at 3 years from diagnosis, or 8% of the initial cohort of 677 patients. LR was recorded in 23 patients, DM in 21 patients, and both LR and DM in 11 patients.

### Late Local Recurrence

Late LR was recorded in 34 of the 377 patients. The median time to late LR was 5 years (mean 5.8; range 3–13) from diagnosis. R0 marginal (HR 3.3; *p* = 0.001) or R1 surgical margin (HR 5.4; *p* = 0.003) and radiotherapy (HR 2.3; *p* = 0.015) revealed statistically significant correlations with late LR using univariable analysis. None of the 19 patients with synovial sarcoma developed late LR (Table 3). When multivariable analysis was performed, R1 (HR 5.0; *p* = 0.005) and R0 marginal (HR 2.6; *p* = 0.02) surgical margins were independently associated with late LR, while radiotherapy was not (Table 4). Age, sex, malignancy grade, histological subtype, tumor site, depth, chemotherapy, and the SING factors (size, vascular invasion, necrosis, and peripheral growth pattern) did not reveal any statistically significant correlation with late LR.

**Table 3:** Univariable Cox proportional hazard model analysis of prognostic factors for late local recurrences

	Patients	Local recurrence	%	HR	95% CI	*p* value	*p* value ^b^
Age (year)							
< 64	183	16	8.7	*Reference*			
≥ 64	194	18	9.3	1.2	0.6–2.4	0.58	0.58
Sex							
Male	214	21	9.8	*Reference*			
Female	163	13	8.0	0.8	0.4–1.6	0.48	0.48
Depth							
Superficial	175	16	9.1	*Reference*			
Deep	196	18	9.2	0.8	0.4–1.7	0.63	0.63
Not available	6	0					
Location							
Lower extremity	233	24	10	*Reference*			
Upper extremity	100	6	6.0	0.6	0.2–1.4	0.25	
Trunk	44	4	9.1	0.9	0.3–2.5	0.82	0.48
Size (cm)							
< 5	144	10	6.9	*Reference*			
≥ 5	229	22	9.6	1.3	0.6–2.8	0.46	0.45
Not available (cm)	4	2					
< 8	244	16	6.6	*Reference*			
≥ 8	129	16	12	1.9	0.9–3.7	0.08	0.08
Not available	4	2					
Malignancy grade							
Low-grade	81	5	6.2	*Reference*			
High-grade	282	28	10	1.6	0.6–4.2	0.32	0.30
Not available	14	1					
Vascular invasion							
No	273	21	7.7	*Reference*			
Yes	38	4	11	1.4	0.5–4	0.55	0.57
Not available	66	9					
Necrosis							
No	157	13	8.3	*Reference*			
Yes	167	14	8.4	1.1	0.5–2.3	0.81	0.81
Not available	53	7					
Growth pattern							
Pushing	20	2	10	*Reference*			
Infiltrative	88	7	8.0	0.8	0.2–3.9	0.76	0.76
Not available	269	25					
Histology							
UPS	78	10	13	*Reference*			
Leiomyosarcoma	81	4	4.9	0.4	0.1–1.4	0.16	
Liposarcoma	47	3	6.4	0.5	0.1–1.9	0.31	
MPNST	18	3	17	1.0	0.3–4	0.90	
Myxofibrosarcoma	68	8	12	0.8	0.5–3.2	0.63	
Synovial sarcoma	19	0	0	–*			
Other	66	6	9.1	0.7	0.3–2.1	0.60	0.15
Surgical procedure ^a^							
Local excision	321	30	9.3	*Reference*			
Amputation	12	0	0	–			
Final surgical margin							
R0 wide	252	13	5.2	*Reference*			
R0 marginal	109	16	15	3.3	1.6–6.8	0.001	
R1	15	4	27	5.4	1.8–16.6	0.003	0.001
Not available	1	1					
Radiotherapy							
No	248	18	7.3	*Reference*			
Yes	129	16	12	2.3	1.2–4.6	0.015	0.017
Chemotherapy							
No	332	30	9.0	*Reference*			
Yes	45	4	8.9	1.4	0.5–4.0	0.55	0.56

*UPS* undifferentiated pleomorphic sarcoma; *MPNST* malignant peripheral nerve sheath tumor

^a^Not applicable to the 44 patients with tumors located to the trunk wall

^b^Overall likelihood ratio test

**Table 4:** Multivariable Cox proportional hazard model analysis of prognostic factors for late recurrence

	Late local recurrence				Late metastasis			
	HR	95% CI	*p* value	*p* value^c^	HR	95% CI	*p* value	*p* value^c^
Size (cm)								
< 5					*Reference*			
≥ 5					2.6	0.9–7.7	0.09	0.09
Not available								
Malignancy grade								
Low-grade					*Reference*^a^			
High-grade					8.3	1.1–61	0.04	0.04
Not available								
Vascular invasion								
No					*Reference*			
Yes					1.1	0.4–2.9	0.82	0.82
Not available								
Final surgical margin								
R0 wide	*Reference*				*Reference*^b^			
R0 marginal	2.6	1.1–6.1	0.02		2.3	1–5.2	0.04	
R1	5.0	1.6–16	0.005	0.009	1.4	0.2–11	0.73	0.065
Not available								
Radiotherapy								
No	*Reference*							
Yes	1.6	0.7–3.4	0.27	0.27				

^a^Vascular invasion was left out when analyzing malignancy grade due to missing values violating the analysis

^b^Tumor size was left out when analyzing R1 intralesional surgical margin due to missing values violating the analysis

^c^Overall likelihood ratio test

The LR were treated with local excision in 29 cases (85%) and amputation in 4 cases (12%). One patient with late LR was not surgically treated due to severe comorbidity.

The LR were identified by the patient in-between clinical visits in 17 cases, on clinical follow-up visits in 7 cases, and in 3 cases by MRI performed of the primary tumor site, due to unspecified local symptoms. Data were not available in 7 cases.

The 3- and 5-year OS from the date of late LR was 77% (95% CI 58–88) and 63% (95% CI 43–78), respectively, compared with 45% (95% CI 36–53) and 40% (95% CI 31–48) for those with an early LR (*p* = 0.01; Fig. 1b).

### Late Metastasis

Late DM was recorded in 32 of the 377 patients. The median time to late DM was 6.2 years (mean 7.1; range 3–13) from diagnosis of the primary tumor. For patients with both late LR and DM, the DM were identified at a median of 31 months (mean 41; range 4–95) after the preceding late LR.

Univariable statistical analysis showed that late DM was associated with high malignancy grade (HR 4.6; *p* = 0.04), size ≥5 cm (HR 3; *p* = 0.003), and vascular invasion (HR 2.9; *p* = 0.01) (Table 5). A statistically significant correlation was also seen with R0 marginal margin (HR 2.2; *p* = 0.04) but not with R1 margin (HR 1.7; *p* = 0.48), resulting in a nonsignificant global significance test (*p* = 0.11). The multivariable analysis revealed that high malignancy grade (HR 8.3; *p* = 0.04) was independently correlated with risk of late DM. This also was observed for R0 marginal margin (HR 2.3; *p* = 0.04), but the global significance test was nonsignificant (*p* = 0.065; Table 4). We could not find any statistical correlations between late DM and age, sex, histological subtype, tumor size dichotomized at 8 cm, tumor site, depth, surgical procedure, tumor necrosis, peripheral growth pattern, radiotherapy, or chemotherapy.

**Table 5:** Univariable Cox proportional hazard model analysis of prognostic factors for late metastasis

	Patients	Metastasis	%	HR	95% CI	*p* value	*p* value^b^
Age (year)							
< 64	183	26	8.7	*Reference*			
≥ 64	194	16	8.2	1.1	0.6–2.3	0.71	0.71
Sex							
Male	214	19	8.9	*Reference*			
Female	163	13	8.0	0.9	0.4–1.8	0.70	0.70
Depth							
Superficial	175	13	7.4	*Reference*			
Deep	196	19	9.7	1.0	0.5–2	0.90	0.90
Not available	6	0					
Location							
Lower extremity	233	21	9.0	*Reference*			
Upper extremity	100	7	7.0	0.8	0.4–2	0.69	
Trunk	44	4	9.1	1.0	0.3–2.9	0.99	0.92
Size (cm)							
< 5	144	5	3.5	*Reference*			
≥ 5	229	25	11	3.0	1.2–7.9	0.03	0.01
Not available (cm)	4	2					
< 8	244	17	7.0	*Reference*			
≥ 8	129	13	10	1.4	0.7–2.8	0.40	0.41
Not available	4	2					
Malignancy grade							
Low-grade	81	2	2.5	*Reference*			
High-grade	282	29	11	4.6	1.1–19	0.04	0.009
Not available	14	1					
Vascular invasion							
No	273	20	7.3	*Reference*			
Yes	38	8	21	2.9	1.3–6.7	0.01	0.02
Not available	66	4					
Necrosis							
No	157	12	7.6	*Reference*			
Yes	167	16	9.6	1.4	0.7–3	0.35	0.35
Not available	53	4					
Growth pattern							
Pushing	20	2	10	*Reference*			
Infiltrative	88	3	3.4	0.4	0.1–2.2	0.27	0.29
Not available	269	27					
Histology							
UPS	78	9	12	*Reference*			
Leiomyosarcoma	81	7	8.6	1.0	0.4–2.7	0.97	
Liposarcoma	47	1	2.1	0.2	0–1.5	0.12	
MPNST	18	3	17	1.2	0.3–4.4	0.80	
Myxofibrosarcoma	68	3	4.4	0.6	0.2–2.2	0.43	
Synovial sarcoma	19	1	5.3	0.4	0.1–3.2	0.40	
Other	66	8	12	1.3	0.5–3.5	0.56	0.32
Surgical procedure^a^							
Local excision	321	27	8.4	*Reference*			
Amputation	12	1	8.3	0.7	0.1–5.5	0.77	0.76
Final surgical margin							
R0 Wide	252	16	6.3	*Reference*			
R0 Marginal	109	14	13	2.2	1.1–4.4	0.04	
R1	15	2	13	1.7	0.4–7.4	0.48	0.11
Not available	1	0					
Radiotherapy							
No	248	21	8.5	*Reference*			
Yes	129	11	8.5	1.4	0.7–2.9	0.37	0.38
Chemotherapy							
No	332	27	8.1	*Reference*			
Yes	45	5	11	2.5	0.9–6.6	0.07	0.10

*UPS* undifferentiated pleomorphic sarcoma; *MPNST* malignant peripheral nerve sheath tumor

^a^Not applicable to the 44 patients with tumors located to the trunk wall

^b^Overall likelihood ratio test

The 3- and 5-year OS from the date of late DM were 42% (95% CI 24–58) and 27% (95% CI 12–44), respectively, compared with 20% (95% CI 15–26) and 11% (95% CI 7–16) for early DM (*p* = 0.01; Fig. 1c).

The late DM were located in the lungs in 14 cases, multiple sites in 6 cases, and lymph nodes in 5 cases. The remaining 7 cases were DM to soft tissue or bone. Nineteen patients had surgery for DM.

## Discussion

The clinical importance of established risk factors to provide prognostic information regarding late recurrences is unclear, and somewhat contradictory results have been provided in previous studies.^18^–^21^ We have analyzed a population-based series of soft tissue sarcomas to evaluate if it is possible to identify patients with an increased risk of late recurrence.

Late recurrence in the literature are often defined as a recurrence diagnosed later than 5 years after the primary diagnosis and treatment.^19^,^20^,^22^ We have chosen 3 years, because we believe it better reflects the recurrence pattern of STS. After 3 years, the incidence of recurrences even out and the hazard rate stays consistent for the years following thereafter (Table 1; Fig. 1a). Moreover, guidelines recommend longer surveillance interval for high-grade tumors to twice a year after 2–3 years, at which time approximately 80% of recurrences have been identified.^3^,^5^,^10^,^14^,^15^,^31^

We found, as expected, that surgical margin correlated with risk of developing late LR. Using R0 wide margin as reference, both R0 marginal and R1 margins were factors that indicated increased risk for late LR. The observed univariable statistical correlation between radiotherapy and increased frequency of late LR, however, is interpreted as being caused by confounding factors, because patients who received radiotherapy, in general, were selected due to inadequate surgical margins and high-risk tumor characteristics.

Surgical margin also correlated with increased risk for late DM, although the global significance test was nonsignificant. An R0 marginal surgical margin correlated with increased risk for late DM, and there was a trend for a correlation of R1, although not statistically significant. The latter is probably explained by a type II error as a result of a small sample size. Tumor size ≥5 cm and vascular invasion had a statistically significant correlation with late DM in univariable analysis but could not be shown to be independent risk factors when performing multivariable analysis. The only risk factor for late DM that remained statistically significant in multivariable analysis was high malignancy grade.

It has been suggested that low-grade tumors may be prone to recur late, but this is not in line with the results in this study.^5^,^32^ Of 100 patients with low-grade tumor, LR occurred in 17 cases of which 5 were late. The primary tumor was excised with R0 wide surgical margin in 3 of these 17 cases. Eight of 100 patients with low-grade tumors developed DM, of whom 6 presented early and 2 presented late. None of these tumors were operated with R0 wide margin, suggesting that patients with low-grade STS treated with R0 wide margin run a low risk for both LR and DM.

Although treatment of recurrent sarcoma may be challenging, we found that the 3- and 5-year OS from diagnosis and treatment of a late LR were comparable to the 3- and 5-year OS for patients after primary diagnosis and treatment of STS.^33^,^34^ Moreover, outcome for patients treated for late recurrences, both LR and DM, was better than for patients treated for early recurrences.

In this study, approximately one-third of late DM were preceded by a late LR. It has been discussed whether the increased incidence of DM following LR is subsequent to the LR itself, but this is now more considered a manifestation of an aggressive tumor biology.^7^,^21^,^35^

Recurrence following a long period of remission after treatment constitutes a clinical problem in various malignancies.^36^ The causing mechanisms are poorly understood, but it is suggested that late recurrence involves multiple mechanisms of tumor dormancy, such as immunosurveillance, angiogenic dormancy, and cellular dormancy (G0–G1 arrest) by disrupted signaling pathways in the microenvironment.^36^,^37^ In this series, STS patients with late events, both LR and DM, had better post-recurrence survival compared with those with early recurrence (Fig. 1b, c) irrespective of tumor histotype. This suggests unknown biological differences between tumors in early and late STS recurrences.

In the literature, reports of surgical margins differ and various margin classification systems are used.^38^ It has been shown that a system that distinguishes marginal margin from wide margin provides more prognostic information than a dichotomous system that only reports negative versus positive margin.^39^–^41^ It has been argued that a marginal margin behaves closer to a positive margin than a negative margin.^39^ We found that R0 marginal margin is correlated with increased frequency of late events compared R0 wide margin, both classified as negative margin.

Early detection of LR and DM may increase chances to offer effective treatment and improve the chance of long-term survival. Studies have shown that approximately one-half of LR are detected by the patient in-between clinical visits, which is in line with our findings.^15^,^42^–^44^ In our series, late LR were identified by the patient in 17 of the 27 cases with available data. In a study by Nakamura et al., approximately one-half of all recurrences (either LR or DM) diagnosed later than 5 years from the initial treatment were detected at follow-up and the other half due to clinical symptoms.^19^ Hence, patient information and awareness of self-examination is an important strategy to improve surveillance as a complement to the routine follow-up.

We were not able to define any specific characteristics of patients or tumors that predict a high risk of late recurrence. Risk factors for late recurrence appear to be the same as for early (supplementary tables), but many risk factors predicting early recurrence did not predict late recurrence. Nonwide surgical margin was associated with increased risk for late recurrence, both LR and DM. We, therefore, emphasize the importance of regular and long-term surveillance for these patients. After treatment of a late LR, it is reasonable to schedule an adequate follow-up strategy, because about one-third of the patients were later diagnosed with DM. High-grade tumors, especially when excised with close margin, should be followed for a long period of time as the risk of late recurrence was not negligible, and the outcome after treatment in this series was good.

This study was performed using data collected prospectively to the national sarcoma quality register during a long period of time. The rarity of late events result in a limited number of cases. This reduces the possibilities to draw strong conclusions and identify clear patterns for risk factors predicting prognosis and outcome. Comparison of results also are made more complicated by international and historical differences in grading systems and resection margins for STS. However, this study is based on a population based series of STS, with long median follow-up and therefore may be considered to reveal a good reflection of incidence and outcome for patients with late recurrence.

In summary, the study confirms that late recurrences of soft tissue sarcoma are relatively rare. Nevertheless, late recurrences do occur and are often successfully treated. Approximately one-third of late DM were preceded by a late LR. Hence, patients should be followed frequently after treatment of a late LR. The better survival after late recurrence might be explained by differences in tumor biology, indicating that early events reflect a more aggressive tumor, even if representing the same histopathologic entity. This calls for an active surveillance strategy of all STS patients with high-grade tumors, because long-term survival indeed is possible to achieve even after a late recurrence.

## Supplementary Information

Below is the link to the electronic supplementary material.

Supplementary file1 (PDF 152 KB)

## References

[1] Jo VY, Fletcher CDM. WHO classification of soft tissue tumours: an update based on the 2013, 4th edn. *Pathology*. 2014;46(2):95-104. 10.1097/PAT.0000000000000050 10.1097/PAT.0000000000000050 24378391

[2] Trovik C, Bauer HCF, Styring E (2017). The Scandinavian Sarcoma Group Central Register: 6,000 patients after 25 years of monitoring of referral and treatment of extremity and trunk wall soft-tissue sarcoma. Acta Orthop..

[3] Zagars GK, Ballo MT, Pisters PWT (2003). Prognostic factors for patients with localized soft-tissue sarcoma treated with conservation surgery and radiation therapy: an analysis of 1225 patients. Cancer..

[4] Gadd MA, Casper ES, Woodruff JM, McCormack PM, Brennan MF (1993). Development and treatment of pulmonary metastases in adult patients with extremity soft tissue sarcoma. Ann Surg..

[5] Casali PG, Abecassis N, Bauer S, et al. Soft tissue and visceral sarcomas: ESMO-EURACAN Clinical Practice Guidelines for diagnosis, treatment and follow-up. *Ann Oncol*. 2018;29:iv51-67. doi:10.1093/annonc/mdy096 10.1093/annonc/mdy096 29846498

[6] Callegaro D, Miceli R, Mariani L, Raut CP, Gronchi A (2017). Soft tissue sarcoma nomograms and their incorporation into practice. Cancer..

[7] Trovik CS, Bauer HCF, Alvegård TA (2000). Surgical margins, local recurrence and metastasis in soft tissue sarcomas: 559 surgically-treated patients from the Scandinavian Sarcoma Group Register. Eur J Cancer..

[8] Cates JMM. The AJCC 8th edition staging system for soft tissue sarcoma of the extremities or trunk: A Cohort study of the SEER database. *JNCCN J Natl Compr Cancer Netw*. 2018;16(2):144-52. 10.6004/jnccn.2017.7042 10.6004/jnccn.2017.7042 29439175

[9] Tanaka K, Ozaki T. New TNM classification (AJCC eighth edition) of bone and soft tissue sarcomas: JCOG Bone Soft Tissue Tumor Study Group. *Jpn J Clin Oncol*. 2019;49(2):103-7. doi:10.1093/jjco/hyy157 10.1093/jjco/hyy157 30423153

[10] Dangoor A, Seddon B, Gerrand C, Grimer R, Whelan J, Judson I (2016). UK guidelines for the management of soft tissue sarcomas. Clin Sarcoma Res..

[11] Carneiro A, Bendahl PO, Engellau J (2011). A prognostic model for soft tissue sarcoma of the extremities and trunk wall based on size, vascular invasion, necrosis, and growth pattern. Cancer..

[12] Gustafson P, Åkerman M, Alvegård TA (2003). Prognostic information in soft tissue sarcoma using tumour size, vascular invasion and microscopic tumour necrosis—The SIN-system. Eur J Cancer..

[13] Sundby Hall K, Bruland ØS, Bjerkehagen B (2018). Adjuvant chemotherapy and postoperative radiotherapy in high-risk soft tissue sarcoma patients defined by biological risk factors—A Scandinavian Sarcoma Group study (SSG XX). Eur J Cancer..

[14] Bone and Soft Tissue Sarcoma of Extremeties and Trunk Wall—Clinical Practice Guidelines. Regional Cancer Centers in Collaboration. https://kunskapsbanken.cancercentrum.se/diagnoser/sarkom/vardprogram/. Accessed 3 June 2020.

[15] Rothermundt C, Whelan JS, Dileo P (2014). What is the role of routine follow-up for localised limb soft tissue sarcomas? A retrospective analysis of 174 patients. Br J Cancer..

[16] Ballo MT, Zagars GK, Cormier JN (2004). Interval between surgery and radiotherapy: effect on local control of soft tissue sarcoma. Int J Radiat Oncol Biol Phys..

[17] Cormier JN, Pollock RE (2004). Soft tissue sarcomas. CA Cancer J Clin..

[18] Lewis JJ, Leung D, Casper ES, Woodruff J, Hajdu SI, Brennan MF (1999). Multifactorial analysis of long-term follow-up (more than 5 years) of primary extremity sarcoma. Arch Surg..

[19] Nakamura T, Grimer RJ, Carter SR, et al. Outcome of soft-tissue sarcoma patients who were alive and event-free more than five years after initial treatment. *Bone Jt J*. 2013;95 B(8):1139-43. doi:10.1302/0301-620X.95B8.31379 10.1302/0301-620X.95B8.31379 23908433

[20] Toulmonde M, Le Cesne A, Mendiboure J (2014). Long-term recurrence of soft tissue sarcomas: prognostic factors and implications for prolonged follow-up. Cancer.

[21] Engellau J, Anderson H, Rydholm A (2004). Time dependence of prognostic factors for patients with soft tissue sarcoma: A Scandinavian Sarcoma Group Study of 338 malignant fibrous histiocytomas. Cancer.

[22] Choy E (2014). Sarcoma after 5 years of progression-free survival: lessons from the French sarcoma group. Cancer.

[23] Bauer HCF, Trovik CS, Alvegård TA (2001). Monitoring referral and treatment in soft tissue sarcoma: study based on 1,851 patients from the Scandinavian Sarcoma Group Register. Acta Orthop Scand..

[24] Styring E, Hartman L, Nilbert M, Rissler P, Rydholm A, von Steyern FV (2014). Small soft tissue sarcomas do metastasize: identification of high-risk tumors. Ann Surg Oncol..

[25] Regionala Cancercentrum i samverkan (Confederation of Regional Cancer Centres). Nationellt kvalitetsregister för sarkom (National Sarcoma Quality Register). https://cancercentrum.se/samverkan/cancerdiagnoser/sarkom/kvalitetsregister/ [Web Page in Swedish]. Accessed 20 Oct 2020.

[26] Oliveira AM, Nascimento AG (2001). Grading in soft tissue tumors: principles and problems. Skeletal Radiol..

[27] Regionala Cancercentrum i samverkan. *Skelett- Och Mjukdelssarkom i Extremiteter Och Bålvägg Nationellt Vårdprogram*.; 2020. https://www.cancercentrum.se/samverkan/cancerdiagnoser/sarkom/skelett-och-mjukdelssarkom/vardprogram/.

[28] Scandinavian Sarcoma Group. *SSG XX*. A Scandinavian Sarcoma Group Treatment Protocol for adult patients with non-metastatic high-risk soft tissue sarcoma of the extremities and trunk wall. 2007. http://www.ssg-org.net/wp-content/uploads/2011/05/SSG-XX-version-June-18-2007-Slutversion-Tryck.pdf.

[29] Enneking WF, Spanier SS, Goodman MA. A system for the surgical staging of musculoskeletal sarcoma. 1980. *Clin Orthop Relat Res*. 2003;(415):4-18. doi:10.1097/01.blo.0000093891.12372.0f 10.1097/01.blo.0000093891.12372.0f 14612624

[30] Trovik CS, Skjeldal S, Bauer H, Rydholm A, Jebsen N. Reliability of margin assessment after surgery for extremity soft tissue sarcoma: the SSG experience. *Sarcoma*. 2012;2012. doi:10.1155/2012/290698 10.1155/2012/290698 PMC3385668 22761544

[31] Sawamura C, Matsumoto S, Shimoji T, Okawa A, Ae K (2014). How long should we follow patients with soft tissue sarcomas?. Clin Orthop Relat Res..

[32] Krieg AH, Hefti F, Speth BM (2011). Synovial sarcomas usually metastasize after >5 years: a multicenter retrospective analysis with minimum follow-up of 10 years for survivor. Ann Oncol..

[33] Karakousis CP (1996). Local recurrence and survival in soft-tissue sarcomas. Ann Surg Oncol..

[34] Ramanathan RC, A’Hern R, Fisher C, Thomas JM (2001). Prognostic index for extremity soft tissue sarcomas with isolated local recurrence. Ann Surg Oncol..

[35] Gustafson P (1994). Soft tissue sarcoma: epidemiology and prognosis in 508 patients. Acta Orthop..

[36] Yeh AC, Ramaswamy S (2015). Mechanisms of cancer cell dormancy-another hallmark of cancer?. Cancer Res..

[37] Aguirre-Ghiso JA (2007). Models, mechanisms and clinical evidence for cancer dormancy. Nat Rev Cancer..

[38] Endo M, Lin PP. Surgical margins in the management of extremity soft tissue sarcoma. *Chinese Clin Oncol*. 2018;7(4):1-14. doi:10.21037/cco.2018.08.10 10.21037/cco.2018.08.10 30173528

[39] Hasley I, Gao Y, Blevins AE, Miller BJ (2018). The significance of a “close” margin in extremity sarcoma: a systematic review. Iowa Orthop J..

[40] Teurneau H, Engellau J, Ghanei I, Vult von Steyern F, Styring E. High recurrence rate of myxofibrosarcoma: the effect of radiotherapy is not clear. *Sarcoma*. 2019;2019. doi:10.1155/2019/8517371 10.1155/2019/8517371 PMC6791216 31662702

[41] Fujiwara T, Stevenson J, Parry M, Tsuda Y, Tsoi K, Jeys L (2020). What is an adequate margin for infiltrative soft-tissue sarcomas?. Eur J Surg Oncol..

[42] Puri A, Gulia A, Hawaldar R, Ranganathan P, Badwe RA (2014). Does intensity of surveillance affect survival after surgery for sarcomas? Results of a randomized noninferiority trial. Clin Orthop Relat Res..

[43] Whooley BP, Gibbs JF, Mooney MM, McGrath BE, Kraybill WG (2000). Primary extremity sarcoma: What is the appropriate follow-up?. Ann Surg Oncol..

[44] Cool P, Grimer R, Rees R (2005). Surveillance in patients with sarcoma of the extremities. Eur J Surg Oncol..

