# Supplementary material for: Late Local Recurrence and Metastasis in Soft Tissue Sarcoma of the Extremities and Trunk Wall: Better Outcome After Treatment of Late Events Compared with Early
Source: Ann Surg Oncol. 2021 Apr 16;28(12):7891–902. doi: 10.1245/s10434-021-09942-8 (PMC8519908; doi:10.1245/s10434-021-09942-8)
Supplement: Supplementary file 1 — Supplementary file1 (PDF 152 KB) [file 10434_2021_9942_MOESM1_ESM.pdf]

Supplementary table 1: Cox proportional hazard model analysis of prognostic factors for early local recurrence of soft tissue sarcoma

				Univariable analysis				Multivariable analysis			
	Patients	Local recurrence	%	HR	95 % CI	p-value	p-value [b]	HR	95 % CI	p-value	p-value [b]
Age											
< 64 years	275	40	15%	*Reference*				*Reference*			
≥ 64 years	402	89	22%	**1.8**	**1.2 - 2.6**	**0.002**	**0.002**	**1.6**	**1.0 - 2.5**	**0.047**	**0.047**
Sex											
Male	369	67	18%	*Reference*							
Female	308	62	20%	1.2	0.8 - 1.7	0.36	0.36				
Depth											
Superficial	267	54	20%	*Reference*							
Deep	402	74	18%	0.9	0.7 - 1.3	0.77	0.77				
Not available	8	1	13%								
Location											
Lower extremity	422	72	17%	*Reference*				*Reference*			
Upper extremity	164	30	18%	1.1	0.7 - 1.7	0.7		0.8	0.5 - 1.3	0.42	
Trunk	91	27	30%	**1.9**	**1.2 - 3.0**	**0.005**	**0.03**	**1.8**	**1.1 - 2.9**	**0.02**	**0.03**
Size											
< 5 cm	194	35	18%	*Reference*							
≥ 5 cm	475	91	19%	1.2	0.8 - 1.7	0.45	0.44				
Not available	8	3									
< 8 cm	373	66	18%	*Reference*							
≥ 8 cm	296	60	20%	1.3	0.9 - 1.8	0.16	0.16				
Not available	8	3									
Malignancy grade											
Low-grade	100	12	12%	*Reference*				*Reference*			
High-grade	558	112	20%	**1.9**	**1.04 - 3.4**	**0.04**	**0.02**	1.2	0.6 - 2.5	0.64	0.63
Not available	19	5									
Vascular invasion											
No	464	81	17%	*Reference*				*Reference*			
Yes	113	29	26%	**1.8**	**1.2 - 2.7**	**0.008**	**0.01**	1.5	0.96 - 2.3	0.07	0.07
Not available	100	19									
Necrosis											
No	226	36	16%	*Reference*							
Yes	368	72	20%	1.4	0.9 -2.0	0.14	0.13				
Not available	83	21									
Growth pattern											
Pushing	26	1	3.8%	*Reference*							
Infiltrative	174	37	21%	6.5	0.9 - 47.2	0.07	**0.01**				
Not available	477	91									
Histology											
UPS	165	43	26%	*Reference*				*Reference*			
Leiomyosarcoma	146	23	16%	**0.5**	**0.3 - 0.9**	**0.01**		**0.5**	**0.3 - 0.8**	**0.006**	
Liposarcoma	70	7	10%	**0.3**	**0.1 - 0.7**	**0.004**		**0.3**	**0.1 - 0.7**	**0.006**	
MPNST	30	3	10%	0.3	0.1 - 1.1	0.07		0.5	0.1 - 1.6	0.24	
Myxofibrosarcoma	112	21	19%	0.6	0.4 - 1.1	0.09		0.6	0.4 - 1.1	0.12	
Synovial sarcoma	43	10	23%	0.7	0.4 - 1.1	0.34		0.9	0.4 - 1.9	0.73	
Other	111	22	20%	0.7	0.4 - 1.1	0.14	0.02	0.8	0.4 - 1.3	0.26	**0.04**
Surgical procedure [a]											
Local excision	561	101	18%	*Reference*							
Amputation	25	1	4.0%	0.2	0.05 - 1.6	0.13	0.047				
Final surgical margin											
R0 Wide	377	41	11%	*Reference*				*Reference*			
R0 Marginal	250	66	26%	**2.9**	**1.9 - 4.2**	**< 0.001**		**2.9**	**1.9 - 4.4**	**< 0.001**	
R1	49	22	45%	**5.6**	**3.3 - 9.4**	**< 0.001**	**< 0.001**	**4.2**	**2.2 - 7.8**	**< 0.001**	**< 0.001**
Not available	1	0									
Radiotherapy											
No	416	87	21%	*Reference*							
Yes	261	42	16%	0.7	0.5 - 1.1	0.11	0.1				
Chemotherapy											
No	586	108	18%	*Reference*							
Yes	91	21	23%	1.2	0.7 - 1.8	0.54	0.55				

Abbreviations: UPS, undifferentiated pleomorphic sarcoma; MPNST, malignant peripheral nerve sheath tumor

[a] Not applicable to the 91 patients with tumours located to the trunk wall.

[b] Overall likelihood ratio test.

Supplementary table 2: Cox proportional hazard model analysis of prognostic factors for early metastasis of soft tissue sarcoma

				Univariable analysis				Multivariable analysis			
	Patients	Metastasis	%	HR	95 % CI	p-value	p-value [b]	HR	95 % CI	p-value	p-value [b]
Age											
< 64 years	275	75	27%	*Reference*							
≥ 64 years	402	115	29%	1.2	0.9 - 1.6	0.18	0.18				
Sex											
Male	369	105	28%	*Reference*							
Female	308	85	28%	1.0	0.8 - 1.4	0.87	0.87				
Depth											
Superficial	267	50	19%	*Reference*				*Reference*			
Deep	402	139	35%	**2.1**	**1.5 - 2.9**	**< 0.001**	**< 0.001**	1.4	0.9 - 2.1	0.09	0.09
Not available	8	1									
Location											
Lower extremity	422	127	30%	*Reference*				*Reference*			
Upper extremity	164	34	21%	**0.7**	**0.6 - 0.97**	**0.03**		0.8	0.5 - 1.2	0.2	
Trunk	91	29	32%	1.1	0.7 - 1.6	0.72	0.06	0.8	0.5 - 1.3	0.31	0.31
Size											
< 5 cm	194	20	10%	*Reference*				*Reference*			
≥ 5 cm	475	167	35%	**4.3**	**2.7 - 6.8**	**< 0.001**	**< 0.001**	**2.1**	**1.2 - 3.6**	**0.006**	**0.006**
Not available	8	3									
< 8 cm	373	66	18%	*Reference*				*Reference*			
≥ 8 cm	296	121	41%	**2.9**	**2.1 - 3.9**	**< 0.001**	**< 0.001**	**1.7**	**1.1 - 2.5**	**0.009**	**0.009**
Not available	8	3									
Malignancy grade											
Low-grade	100	6	6.0%	*Reference*				*Reference*			
High-grade	558	183	33%	**6.7**	**3.0 - 15.2**	**< 0.001**	**< 0.001**	**3.2**	**1.2 - 8.3**	**0.02**	**0.02**
Not available	19	1									
Vascular invasion											
No	464	108	23%	*Reference*				*Reference*			
Yes	113	64	57%	**3.6**	**2.6 - 4.9**	**< 0.001**	**< 0.001**	**2.4**	**1.7 - 3.3**	**< 0.001**	**< 0.001**
Not available	100	18									
Necrosis											
No	226	30	13%	*Reference*				*Reference*			
Yes	368	143	39%	**3.7**	**2.5 - 5.5**	**< 0.001**	**< 0.001**	**1.8**	**1.1 - 2.8**	**0.02**	**0.02**
Not available	83	17									
Growth pattern											
Pushing	26	4	15%	*Reference*							
Infiltrative	174	54	31%	2.3	0.8 - 6.4	0.1	0.07				
Not available	477	132									
Histology											
UPS	165	50	30%	*Reference*				*Reference*			
Leiomyosarcoma	146	46	32%	1.0	0.7 - 1.5	0.97		1.1	0.7 - 1.7	0.64	
Liposarcoma	70	13	19%	**0.5**	**0.3 - 0.9**	**0.03**		0.5	0.3 - 1.1	0.09	
MPNST	30	10	33%	1.1	0.5 - 2.1	0.86		1.8	0.9 - 3.6	0.1	
Myxofibrosarcoma	112	21	19%	**0.6**	**0.3 - 0.9**	**0.03**		0.6	0.3 - 1.0	0.06	
Synovial sarcoma	43	21	49%	1.5	0.9 - 2.5	0.13		1.2	0.7 - 2.2	0.52	
Other	111	29	26%	0.8	0.5 - 1.3	0.38	**0.01**	1.2	0.7 - 2.1	0.6	**0.04**
Surgical procedure [a]											
Local excision	561	150	27%	*Reference*				*Reference*			
Amputation	25	11	44%	**1.9**	**1.05 - 3.5**	**0.04**	0.06	1.7	0.8 - 3.5	0.19	0.18
Final surgical margin											
Wide	377	93	25%	*Reference*				*Reference*			
Marginal	250	78	31%	**1.4**	**1.04 - 1.9**	**0.03**		**1.5**	**1.0 - 2.3**	**0.04**	
Intralesional	49	19	39%	**1.9**	**1.2 - 3.1**	**0.01**	**0.01**	1.7	0.8 - 3.4	0.16	**0.09**
Not available	1	0									
Radiotherapy											
No	416	90	22%	*Reference*				*Reference*			
Yes	261	100	38%	**1.9**	**1.4 - 2.6**	**< 0.001**	**< 0.001**	1.0	0.7 - 1.5	0.95	0.95
Chemotherapy											
No	586	154	26%	*Reference*				*Reference*			
Yes	91	36	40%	**1.5**	**1.0 - 2.1**	**0.03**	**0.04**	**0.6**	**0.4 - 1.0**	**0.046**	0.046

Abbreviations: UPS, undifferentiated pleomorphic sarcoma; MPNST, malignant peripheral nerve sheath tumor

[a] Not applicable to the 91 patients with tumours located to the trunk wall.

[b] Overall likelihood ratio test.
